# Percutaneous Transhepatic Bile Duct Ablation with n-Butyl Cyanoacrylate in the Treatment of a Biliary Complication after Split Liver Transplantation

**DOI:** 10.1155/2009/824803

**Published:** 2009-06-24

**Authors:** Andrea Lauterio, Abdallah Slim, Paolo Aseni, Alessandro Giacomoni, Stefano Di Sandro, Rocco Corso, Iacopo Mangoni, Plamen Mihaylov, Mohammed Al Kofahi, Vincenzo Pirotta, Luciano De Carlis

**Affiliations:** ^1^Division of General Surgery and Abdominal Organ Transplantation, Niguarda Hospital, 20162 Milan, Italy; ^2^Division of Interventional Radiology, Niguarda Hospital, 20162 Milan, Italy

## Abstract

Biliary complications continue to be a major cause of morbidity after split-liver transplantation (SLT). In this report we describe an uncommon late biliary complication. One year after SLT the patient showed an intrahepatic bile dicy dilatation with severe cholangitis episodes. The segmentary bile duct of hepatic segment VI-VII draining in the left duct was unidentified and tied at the time of the in situ split-liver procedure. We perform a permanent obliteration of the dilated intrahepatic ducts by a percutaneous embolization using an n-butyl cyanoacrylate (NABC). The management of biliary complications after SLT requires a multidisciplinary approach. The use of NBCA in obliteration of a dilated bile duct seems to be a safe procedure with good results providing a less invasive option than hepatic resection and decreasing the morbidity associated with chronic external biliary drainage. Further studies are needed to determine whether this approach is effective and safe and whether it could reduce hospital stay and cost.

## 1. Introduction

Split-liver transplantation (SLT) is an attractive alternative procedure to expand the donor pool in patients waiting for liver transplantation. Paramount to the success of SLT is a careful donor and recipient selection. The standard split-liver procedure for a child and an adult (Adult/Pediatric Split Liver Ttransplantation, A/P SLT), by splitting segment II-III for pediatric recipient and segment I-IV-V-VI-VII-VIII for an adult, is an accepted surgical option with good results both for the adult and for pediatric recipient [1].

Biliary complications continue to be the major cause of morbidity after SLT with a reported high incidence ranging between 10% and 32% [1, 2].

As a consequence of bile duct anatomic variations, SLT requires a precise knowledge of the liver anatomy [3, 4]. The challenge of this procedure is represented by a preoperative radiological assessment of the biliary anatomy often unavailable at the donor's hospital. Thus the risk of biliary duct injury during the splitting procedure is usually considered higher than during living donor procedure. 

In this report we describe an uncommon late biliary complication that occurred after SLT and was successfully treated by a multidisciplinary approach. 

## 2. Case Report

A 63-year-old male with hepatitis C-related cirrhosis was referred for liver transplantation to our institution. We performed a conventional A/P SLT with in situ technique providing the left lateral segment for a child (segments II-III) and leaving the right lobe graft (segment I-IV-V-VI-VII-VIII) for an adult recipient. The celiac trunk was left on the left graft while the right hepatic artery remained on the right graft. The common hepatic bile duct was left on the right graft. The patient was transplanted using the piggy-back technique without a veno-venous bypass. The biliary tract was reconstructed performing a duct-to-duct anastomosis using a T-tube by our standard technique previously described [5].

**Figure 1:**
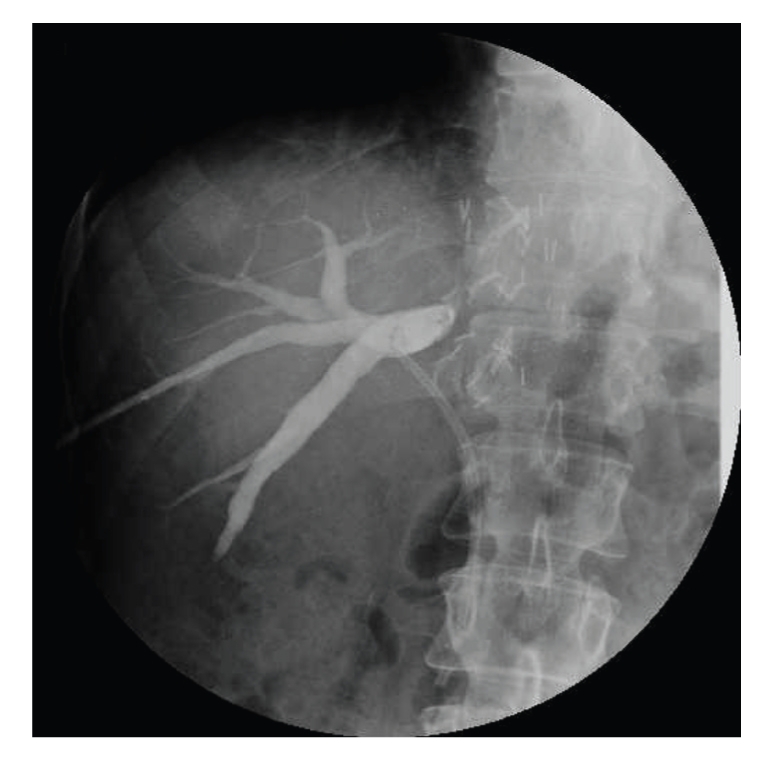
Percutaneous transhepatic cholangiography (PTC) showing the dilated segmental intrahepatic ducts excluded by the main biliary duct. A plastic stent is placed in the main duct.

**Figure 2:**
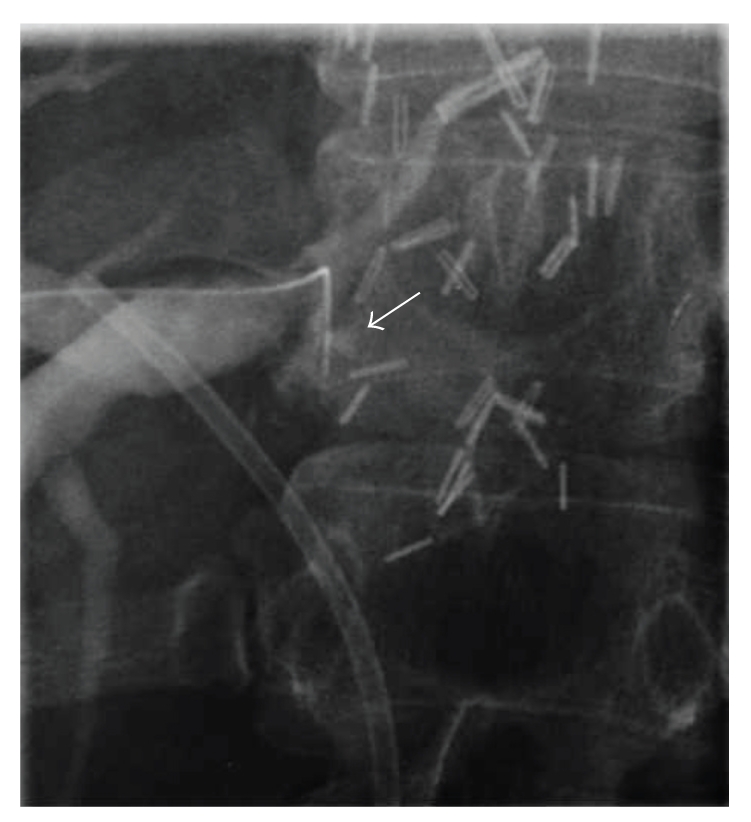
Cholangiogram showing the guide wire (arrow) during unsuccessful attempts to cross the anastomotic obstruction.

A cholangiography through the T-tube was performed on postoperative day 14, and the T-tube was clamped before patient discharge. Three months after A/PSLT the T-tube was removed after a cholangiography with normal findings. One year after transplant the patient showed abnormal liver function tests, hyperbilirubinemia, leukocytosis, and elevated g-glutamyl transpeptidase (GGT), and mild elevation in alanine transaminase (ALT). The patient underwent a doppler ultrasound that showed (a patent hepatic artery) an intrahepatic bile duct dilatation and an anastomotic biliary stricture. These findings were confirmed by a magnetic resonance cholangiography (MRC). The anastomotic stricture was treated by stenting the main biliary duct during an endoscopic retrograde cholangiopancreatoghaphy (ERCP) without any evidence of intrahepatic biliary dilatation. After this procedure the patient was submitted to a percutaneous transhepatic cholangiography (PTC) showing a complete obstruction of segments VI and VII biliary branches near the duct-to-duct biliary anastomosis (Figure 1). A percutaneous biliary drain was left inside the distended biliary branches. The patient was discharged leaving the external biliary drain open allowing bile drainage and an easy access for repeated radiologic treatment and an internal stent in the common bile duct. Three months later the patient underwent a surgical revision because of repeated episodes of cholangitis. During surgery an intraoperative cholangiography was performed through the biliary drain confirming a bile duct dilatation at the level of segments VI and VII. It was impossible to cross the biliary stricture by a torque catheter and by hydrophilic guide wire (Figure 2).

We supposed that the segmentary bile duct branch of posterior segments VI and VII draining in the left bile duct was unidentified and tied at the time of the in situ split-liver procedure during the parenchymal transection. A primary biliary reconstruction by biliary repair or biliodigestive anastomosis was considered at high risk because of the presence of postsurgical adhesions sand due to fibrotic tissue replacing the parenchymal transected area. A liver resection of the dilated segments VI and VII was considered at high risk of leaving an inadequate liver mass. 

**Figure 3:**
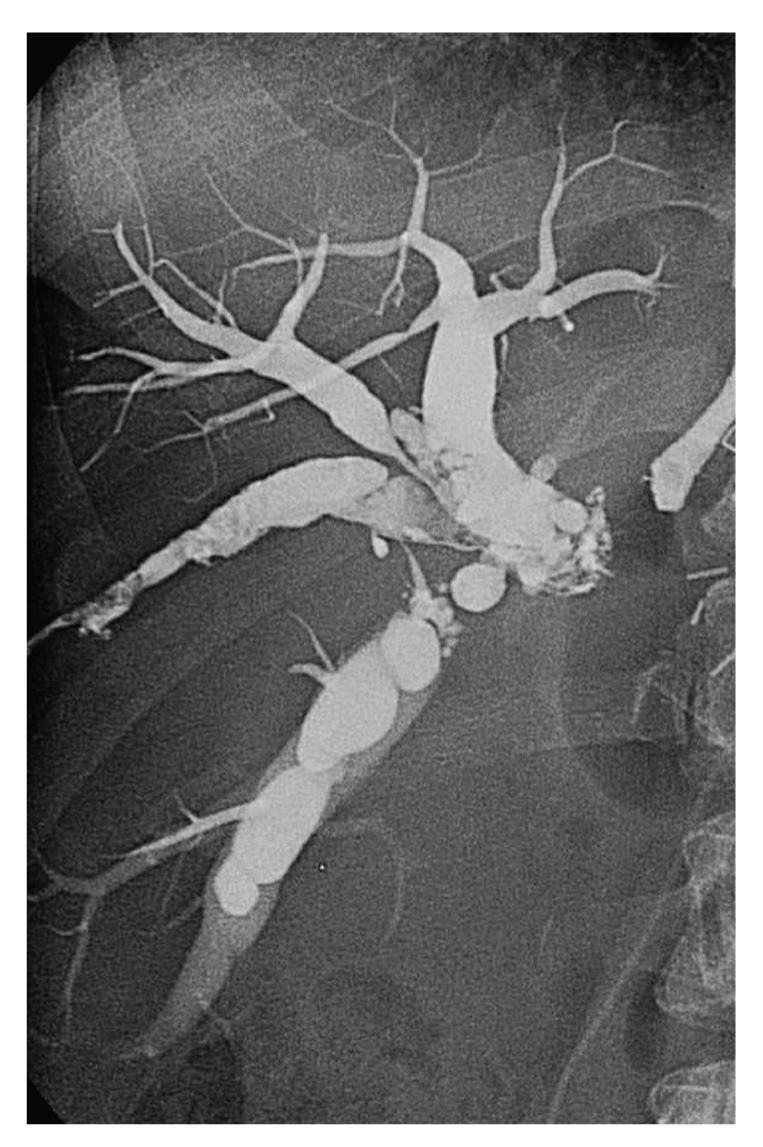
Intraoperative cholangiogram showing the complete filling with glue of excluded intrahepatic bile ducts.

We decided to perform a permanent intraoperative obliteration of the dilated intrahepatic ducts by a percutaneous embolization using a nonresorbable agent. With a fluoroscopic guidance through the transhepatic access we positioned a 5-French polyethylene catheter inside the ducts, preliminary flushed by a nonionic dextrose solution. We then injected the tissue adhesive agent n-butyl cyanoacrylate (NBCA, Glubran 2, GEM, Viareggio, Italy) mixed with ionized oil (Lipiodol, Guebert, Aulnay-sous-Bois, France) for opacization in a ratio of 1:5. This solution completely filled the biliary duct, and the occlusion was totally accomplished in a few seconds (Figure 3).

During the first 3 days after the chemical bile duct embolization, the patient had a low fever with a slightly abnormal liver function test. A computed tomography (CT) scan performed 6 months later showed no sign of hepatic abscesses, and the bile duct dilatation was completely occupied by the NBCA-Lipiodol mixture. One year after the procedure patient showed normal liver function tests without no episodes of cholangitis.

## 3. Discussion

The management of biliary complications after liver transplantation requires a multidisciplinary approach.

Chemical bile duct embolization treatment could represent a valuable solution to treat uncommon biliary complications. These tissue adhesive glues are low-viscosity liquid monomers that undergo rapid polymerization and solidification when they come into contact with organic fluids such as bile. NBCA is a permanent liquid embolic material that produces long-term occlusion in vessels of various size through an inflammatory tissue response resulting in vessel thrombosis or tissue atrophy.

Little is known about the use of cyanoacrylate compounds, and unlike European countries the use of Glubran has not been approved by the Food and Drug Administration yet. 

Other authors have described the efficacy of biliary duct ablation by NBCA in patients with persistent postsurgical bile leaks after lobectomy or cholecystectomy. Vu et al. treated six patients with persistent postsurgical bile leaks as a complication after hepatic lobectomy or cholecistectomy using NBCA glue for the obliteration of isolated segmental bile ducts in four cases [6]. Endoscopic treatment of biliary leakage by NBCA has been described by Seewald in nine patients in whom primary stent placement or nasobiliary drain was unsuccessful [7]. More recently, Romano et al. [8] described the use of a cyanoacrlylate in the treatment of a pancreatic fistula after distal pancreatectomy.

The percutaneous interventional technique represents an effective valuable approach to reduce mortality and morbidity in the treatment of biliary complications after liver transplantation.

The use of NBCA in obliteration of a dilated bile duct seems to be a safe procedure with good results providing a less invasive option than hepatic resection above all in high-risk patients with posttransplant bile duct injuries, decreasing the morbidity associated with chronic external biliary drainage.

Futher studies are needed to determine whether this approach is effective and safe and whether it could reduce hospital stay and costs.

## Abbreviations

SLT: Split-liver transplantation
GGT: g-glutamyl transpeptidase
ALT: Alanine transaminase
MRC: Magnetic resonance cholangiography
PTC: Percutaneous transhepatic cholangiography
NBCA: n-butyl cyanoacrylate

## References

[1] Rogiers X, Sieders E (2008). Split-liver transplantation: an underused resource in liver transplantation. *Transplantation*.

[2] Renz JF, Yersiz H, Reichert PR (2003). Split-liver transplantation: a review. *American Journal of Transplantation*.

[3] Onishi H, Kawarada Y, Das BC (2000). Surgical anatomy of the medial segment (S4) of the liver with special reference to bile ducts and vessels. *Hepato-Gastroenterology*.

[4] Chaib E, Bertevello P, Saad WA, Pinotti HW, Gama-Rodrigues J (2007). The main hepatic anatomic variations for the purpose of split-liver transplantation. *Hepato-Gastroenterology*.

[5] Belli L, De Carlis L, Del Favero E (1991). Biliary complications in orthotopic liver transplantation: experience with a modified technique of duct-to duct reconstruction. *Transplant International*.

[6] Vu DN, Strub WM, Nguyen PM (2006). Biliary duct ablation with n-butyl cyanoacrylate. *Journal of Vascular and Interventional Radiology*.

[7] Seewald S, Groth S, Sriram PVJ (2002). Endoscopic treatment of biliary leakage with n-butyl-2 cyanoacrylate. *Gastrointestinal Endoscopy*.

[8] Romano A, Spaggiari M, Masetti M (2008). A new endoscopic treatment for pancreatic fistula after distal pancreatectomy: case report and review of the literature. *Gastrointestinal Endoscopy*.

